# Double-way spectral tunability for the control of optical nanocavity resonance

**DOI:** 10.1038/srep17907

**Published:** 2015-12-08

**Authors:** Fadi I. Baida, Thierry Grosjean

**Affiliations:** 1 Département d’Optique P.M. Duffieux, Institut FEMTO-ST, UMR 6174 CNRS, Université Bourgogne Franche–Comté, 15B Avenue des Montboucons, 25030 Besançon Cedex, France

## Abstract

Scanning Near-field Optical Microscopy (SNOM) has been successful in finely tuning the optical properties of photonic crystal (PC) nanocavities. The SNOM nanoprobes proposed so far allowed for either redshifting or blueshifting the resonance peak of the PC structures. In this paper, we theoretically demonstrate the possibility of a redshifting (up to +0.65 nm) and a blueshifting (up to −5 nm) the PC cavity resonance wavelength with a single perturbation element. As an example, a fiber bowtie-aperture nano-antenna (BNA) engraved at the apex of a SNOM tip is proposed to play this role. The double-way tunability is the result of a competition between an induced electric dipole (BNA at resonance) leading to a redshift and an induced magnetic dipole (the tip metalcoating) giving rise to a blueshift of the resonance wavelength. We demonstrate that the sign of the spectral shift can be simply controlled through the tip-to-cavity distance. This study opens the way to the full postproduction control of the resonance wavelength of high quality-factor optical cavities.

It is known that for a given optical cavity, the resonance properties depend on the volume allocated to the excited mode. This property generated several recent studies that deal with the resonance wavelength (RW) control especially in the case of high quality-factor resonators such as photonic crystal cavities. Modifications of this volume can be achieved through different physical processes. Thus, optical^1^,^2^, electrical^3^,^4^, chemical^5^,^6^ or mechanical^7^,^8^ effects were exploited to shift the RW by slightly modifying the cavity resonance properties. Plasmonic^9^ and, more widely, any Fano-type resonances are also ideal candidates for such control.

In all cases, the perturbation must be small enough not to cancel the cavity resonance. Basically, the cavity mode volume can be modified by immersing a small dielectric element within the evanescent field of the cavity^10^,^11^,^12^,^13^,^14^. Thereby, the effective volume of the latter is increased (light penetrates inside the dielectric) leading to a redshift of its RW. Contrarily, if we replace dielectric by metal that inhibits the light permeation, the electromagnetic field will be mostly squeezed so that the whole mode volume decreases. The direct consequence is then a blueshift of the RW^2^,^15^. Nonetheless, if the metallic structure exhibits localized resonance (almost with dipolar behavior), redshifts may occur after complete compensation of the mode squeezing due to the presence of the metal as already demonstrated in reference^9^. Generally, for these effects which are opposed, two cases are possible: either they are compensated, either one of them is dominant such that we can not discern them^16^. In all cases, the overall frequency shift is so small (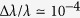) that resonators of very large Q-factors are needed^17^ for experimental evidence. Thus, the question is how to control the contribution of each effect in order to actively control the RW? A basic idea consists of the design of an element that allows the two above cited effects and also permits adjusting the influence of each one through its interaction with the optical cavity. Nevertheless, the interaction of the cavity with this element should be as weak as possible to not completely break down the cavity resonance.

Appropriately, to highlight the existence of these two effects within a single configuration, some authors proposed a metal coated SNOM tip with small aperture at its apex in order to control the resonance wavelength of an optical cavity^16^. Such a perturbation element combines, at least, an induced electric dipole together with a magnetic one. Unfortunately, the cavity-tip interaction was dominated by the magnetic contribution due to the large metal volume of the tip apex that is involved in the interaction. Nevertheless, theoretical calculations based on FDTD numerical simulations showed that it is possible to get both red (very weak shift of only 30*pm*) and blueshifts (almost 2.8 Å) with the same SNOM tip by placing its apex at specific positions in the vicinity of the cavity. Thus, the discrimination between the two effects was done spatially by exciting selectively the electric or the magnetic dipolar response of the tip through the knowledge of the electromagnetic field near the cavity.

As known, the magnetic effect, that is induced by the longitudinal component of the magnetic field (here along the tip axis) and leads to a blueshift, can be seen as a consequence of the cavity mode squeezing by the metallic apex of the tip. The electric effect of the tip apex is induced by the transversal component of the electric field around the tip apex and leads to a redshift of the resonance wavelength that can be seen to be due to an increase of the cavity mode volume. Based on refs 10,16,18, the contribution of the electric and magnetic dipoles to the wavelength shift can be written as:


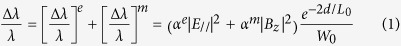


where *α*^*e*^ and *α*^*m*^ are electric and magnetic polarizabilities of the BNA-on-tip respectively, *E*_//_ is the transverse component of the electric field within the unperturbed cavity, *B*_*z*_ is the magnetic longitudinal component at the top of the unperturbed cavity and *W*_0_ is the total stored energy in the PC cavity at resonance. The decay term 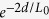 is the same for both magnetic and electric field and allows the determination of the fields at the tip apex position (at a distance *d* from the cavity) through the knowledge of the decay length *L*_0_. The *α*^*e*^ and *α*^*m*^ terms have an opposite sign and both depend on the tip apex (the nature and the volume of metal that is involved by the tip-PC interaction for the magnetic term *α*^*m*^ and the size and shape of the aperture where the electric effect takes place for the electric term *α*^*e*^). Consistently with the results of ref. 16 and with recent theoretical and experimental investigations^19^,^20^, a predominant contribution (from 2.5:1 to 10:1) of the magnetic effect was demonstrated compared to the electric one in the case of circular hole aperture engraved in metallic film.

In order to bypass this prevalence of the magnetic effect, a perturbation element that exhibits electric resonance would be necessary to reinforce the electric effect of the tip. More precisely, this resonance must exhibit a small quality factor in order not to interact destructively with the optical cavity. Gap-based nano-antennas are ideal candidates due to their optical resonance that gives rise to spatially confined electric field over a broad spectral range (quality factor of around 3)^21^.

In this paper, we propose an example of such configuration where an efficient resonator having quite large quality factor, a photonic crystal cavity, is coupled to a bowtie nano-aperture antenna (BNA) engraved at the end of a SNOM tip (called BNA-on-tip in the following). Due to the electric resonance of the BNA, the value of the ratio 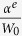 is not only enhanced but it depends itself on the distance between the tip and the cavity. In fact, when the BNA resonates, the interaction between the cavity and the BNA-on-tip can significantly decrease the stored energy (*W*_0_) in the cavity due to a funneling effect^22^ towards the BNA that dissipates a significant part of the energy by both scattering and absorption. In this case, the quasi-static approximation (a fortiori the depolarization regime^23^) is not fulfilled because the size of the BNA-on-tip apex cannot be considered as larger than its skin depth at resonance due to the light funneling phenomenon toward the BNA. Analytic expression of *α*^*e*^ can then hardly be derived contrarily to the case of quasi-static (QS) approximation. In fact, as it will be demonstrated in the following, the QS approximation that leads to a constant value of *α* terms is only fulfilled when the BNA is off-resonance i.e. when the electric effect is canceled (only *α*^*m*^ is constant). Moreover, at the BNA resonance, the coupling between the two resonators greatly depends on the distance between them and thus the electromagnetic field above the PC does not undergo a basic evanescent decay as described in Eq. 1. Indeed, a critical coupling between the two resonators can take place for specific value of the BNA-to-PC distance^21^,^24^. This sensitivity to the distance *d*, which will be presented in a detailed way in the following, is exploited to control the weight of the tip electric effect with respect to the magnetic one onto the PC cavity.

## The proposed structure

As mentioned previously, the structure is composed of a BNA tip coupled to a photonic crystal cavity. Before the study of their coupling, we will present the properties of each one.

### a) Study of the BNA

**Figure 1:**
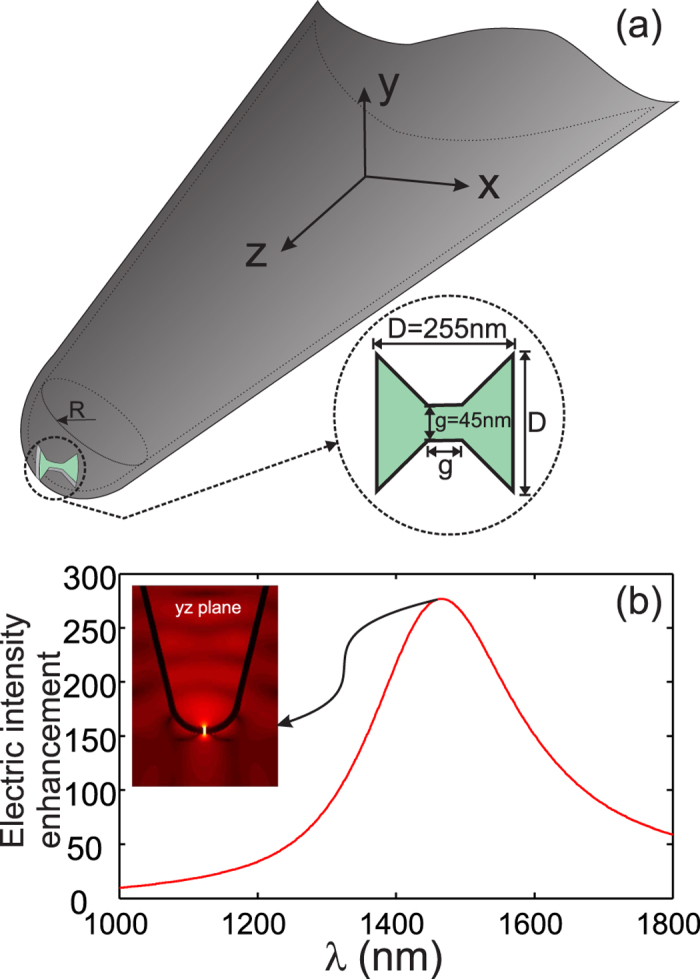
(**a**) Schematic of the BNA engraved at the tip apex of radius *R*. (**b**) Enhancement factor of the BNA-on-tip measured 15 nm in front of its apex and defined as the ratio of the intensity with BNA by the same intensity calculated without metal coating. The inset of (**b**) shows the intensity distribution (logarithmic scale) in the *yOz* longitudinal plane to point out the light confinement occurring in the gap zone.

The BNA was designed through its geometrical parameters (see Fig. 1a) to obtain a resonance wavelength close to the one of the PC cavity. Thus, on the basis of ref. 25, a metal coated SNOM tip with 100 *nm* thick aluminum layer and an apex radius *R* is considered to receive the BNA. The latter is supposed to be centered on the tip axis and has a gap of *g* = 45 *nm* and a side width of *D* = 255 *nm* (see Fig. 1a). The near-field enhancement factor, calculated by 3D-FDTD home-made code, is presented in Fig. 1b in addition to the spatial distribution of the electric field intensity in the *yOz* longitudinal plane at its resonance (inset of the same figure). The spectrum given in Fig. 1b is calculated at 15 *nm* in front of the BNA-on-tip when it is illuminated by the fundamental guided mode of the optical fiber. Normalization of this near-field signal by the same intensity calculated without the BNA (uncoated tip) leads to an enhancement factor that reaches 280 at resonance.

As clearly seen from the inset of Fig. 1b, the resonant BNA acts as a light concentrator. Note that this resonance is only obtained for the electric field component that is parallel to the metallic arms of the BNA (*Oy* direction here). The resulting polarization sensitivity of the BNA allows distinction between the two transversal electric field components^22^,^25^ and will be exploited for the discrimination between the magnetic effect of the electric one. Let us emphasize that this resonance corresponds to the excitation, at its cutoff wavelength, of the BNA fundamental guided mode that propagates through the metal thickness^26^. For a given metal, this cutoff wavelength can be analytically expressed as a function of the BNA geometrical parameters as demonstrated in ref. 26 allowing a very simple design of the BNA.

### b) Study of the photonic crystal cavity

**Figure 2:**
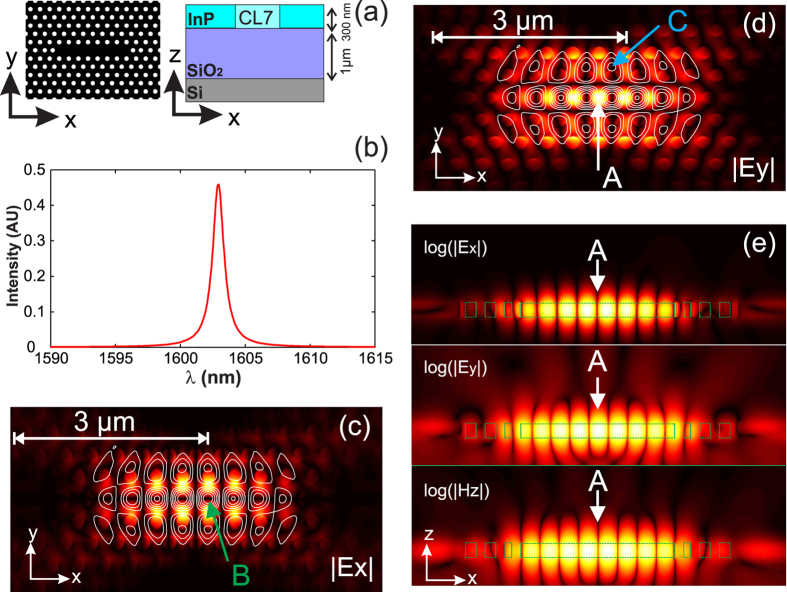
Schematic of the proposed CL7 cavity in (**a**) with vertical and horizontal cross sections along the structure. The near-field spectrum, calculated at the center of the cavity, is presented in (**b**) where the resonance of the fundamental mode is shown to occur at 
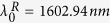
 with a quality factor of *Q*_*CL*7_ = 1590. The amplitude of the two transversal electric field components at this resonance are given in the *xOy* plane located 15 *nm* above the InP membrane in (**c**,**d**). The white lines are iso-amplitude contours of the vertical component of the magnetic field (*H*_*z*_) (**e**) Spatial distributions of the two transversal electric field and the longitudinal magnetic field components (logarithmic scale) at resonance in a vertical plane (*xOz*) passing by the center of the CL7.

Let us now consider the photonic crystal -based optical cavity. It is composed of seven missed and aligned air holes (cavity is then named CL7) in a triangular lattice photonic crystal that was designed to exhibit confined in-plane Bloch modes (period *a* = 420 *nm* and hole radius *r* = 0.25*a*)^27^. In addition, the two air holes located at the edges of the cavity are slightly moved out from the cavity in order to enhance the quality factor of its fundamental mode^28^. The PC is made in a InP layer of 300 *nm* thickness deposited on a 1 *μm* silica layer separating the InP from silicon substrate (see Fig. 2a). The fundamental mode of the cavity cannot be excited by propagating incident wave since it is spectrally located under the light line in the dispersion diagram of the PC structure.

Therefore, the excitation of the CL7 cavity is simulated with a 3D-FDTD homemade code by considering a point source (dipole) emitting over a wide spectral range and positioned in the vicinity of the cavity so that its orientation and position agree with the excitation of the cavity fundamental mode. The dipole source has a physical meaning since it can model a quantum emitter coupled to the cavity^29^. This coupling leads to the excitation of several cavity modes, thus, we have restricted our spectral study to a small spectral range centered on the resonance wavelength of the fundamental mode of the cavity. The near field spectral density of the CL7 cavity is shown on Fig. 2b and exhibits a RW equal to 
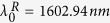
 with a quality factor estimated to *Q*_*CL*7_ = 1590.

3D-FDTD results giving the spatial distributions, at 15 *nm* above the PC cavity, of the transversal electric field components (*E*_*x*_ and *E*_*y*_) are shown in Fig. 2c,d while the longitudinal magnetic field component (*H*_*z*_) is given by the white contour plots on the same figures. On the first hand, one can see from these two figures that the transversal (x and y) components are always spatially interleaved so that the *E*_*x*_ component vanishes when the *E*_*y*_ one is maximum. This specific property is of interest for the demonstration of the RW tunability in our case. In other words, when the BNA is placed above the center of the cavity (A position in Fig. 2c), its state (on- or off-resonance) can be simply controlled through its direction: on-resonance if the BNA’s metallic arms are along the *y*-direction and off-resonance when they are along the *x*-direction, without changing the BNA-on-tip spatial position^22^. This allows here for differentiating the interaction effect induced by the BNA resonance (dielectric term) from that of the tip metal coating (magnetic response). From Fig. 2e, that shows the spatial distribution of the two transverse electric field components and the longitudinal magnetic one in the *xOz* vertical plane, we estimated the value of the decay length to be *L*_0_ = 98 *nm*. We verified that, both electric and magnetic fields have the same decay length.

### Study of the coupling

The whole structure (BNA-on-tip + CL7) is numerically studied with a 3D-FDTD homemade code that incorporates non-uniform mesh together with a sub-griding technique in order to faithfully describe the structure. First, the BNA-on-tip is supposed to be centered on the CL7 cavity. According to Fig. 2c,d, only the *E*_*y*_ component of the electric field exists at this location (A), *E*_*x*_ is equal to zero. Consequently, the BNA metallic arms must be oriented along the *y*-direction to induce its resonance.

**Figure 3:**
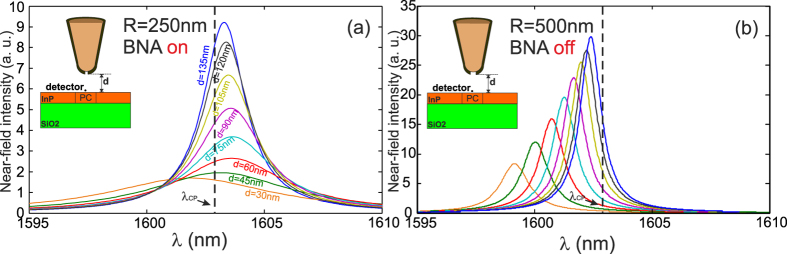
Near field spectra calculated 15 *nm* in front of the PC for two different BNA-on-tip: in (**a**) the BNA resonates meaning that its metallic arms are along the *y*-direction. In addition, the tip radius is fixed to *R* = 250 *nm* in order to minimize the blueshift that directly depends on the tip size. Consequently, blueshift only appears for a small distance *d* = 30 *nm* while the BNA resonance is responsible for the redshift because a part of the light penetrates inside the gap leading to a larger mode volume. In (**b**) the radius of the tip is increased to *R* = 500 *nm* to enhance the mode squeezing and the BNA is rotated by 90° in order to inhibit its resonance. In this case, the BNA-on-tip almost plays the role of a metallic rod as in ref. 2 leading only to a blueshift whatever the value of the distance *d* is as experimentally demonstrated in^15^.

Optical near-field spectra calculated 15 *nm* above the center of the CL7 are presented in Fig. 3a for different values of the BNA-to-CL7 distance (*d*) when the BNA is oriented along the *Oy* direction (BNA on-resonance) and for a tip apex of *R* = 250 *nm*. We recall that *R* is the dielectric tip apex radius before the metal coating). One can clearly see form Fig. 3a the occurrence of two opposite behaviors: a significant redshift for large value of *d* and a small blue one for *d* = 30 *nm*. Thus, two different phenomena are in competition: the metallic tip effect or magnetic effect that squeezes the PC cavity mode (blueshift) and the BNA resonance that tends to increase the mode volume by extending the light distribution inside its gap (dielectric resonance). This interpretation is clearly confirmed thanks to the results of Fig. 3b obtained for off-resonant BNA and showing, similarly to reference^15^, only blueshifts whatever the distance separating the tip from the cavity. However, the quality factor of these resonances evolves differently depending on the BNA state (on or off) as it will be seen in the following.

**Figure 4:**
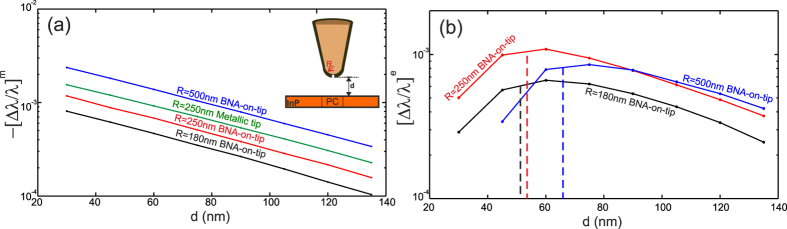
Magnetic in (**a**) and electric in (**b**) relative shifts of the resonance wavelength of the CL7 fundamental mode as a function of the BNA-to-CL7 distance *d*. Three different BNA-on-tip apex radius are considered (*R* = 180 *nm* in black, *R* = 250 *nm* in red and *R* = 500 *nm* in blue). The magnetic shift induced by an apertureless SNOM tip with radius *R* = 250 *nm* is also presented in green line in (a).

Second, in order to quantify the magnetic and the electric effects, we have numerically studied the coupling for different values of the tip radius in both on- and off-resonant BNA cases. Figure 4a shows the variations of the relative shift as a function of the BNA-to-PC distance for off-resonant BNA when the tip is placed above the center of the CL7 cavity (point A indicated by the white arrow in Fig. 2d). Note that at this location, the longitudinal magnetic field vanishes (see white contours) and the obtained magnetic effect is due to the interaction of the tip sidewalls with the closest lobes of the magnetic field. In this case, the electric term of Eq. 1 is negligible and only magnetic coupling occurs between the BNA-on-tip and the PC (second term of Eq. 1). The linear behaviors observed in Fig. 4a confirm that the magnetic effect can be derived from a quasi-static (QS) approximation as in ref. 16 whatever the value of the tip apex radius (from *R* = 180 *nm* to *R* = 500 *nm*).

On the other hand, the slope of the four lines in Fig. 4a, which is equal to 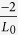 is in a excellent agreement with the decay length *L*_0_ = 98 *nm* of the electromagnetic field above the unperturbed cavity. Values of *L*_0_ = 108 *nm*, 105 *nm* 104 *nm* and 102 *nm* are obtained for *R* = 500 *nm*, 250 *nm*, 300 *nm* and 180 *nm* respectively. Once again, the QS approximation is validated in the case of off-resonant BNA. Nevertheless, the small increasing in the decay length with respect to the tip radius can be attributed to the longitudinal component of the magnetic field (*B*_*z*_) that increases with respect to the unperturbed cavity due to the coupling with the BNA-on-tip. Nonetheless, we have calculated the cavity energy *W*_0_ and determine the effective magnetic polarizability for the four values of the radius using Eq. 1. The obtained values are: 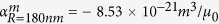, 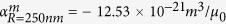, 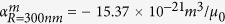 and 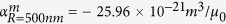. These values well agree with the value (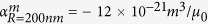) obtained in ref. 16 for a cylindrical aperture metal coated SNOM tip with a radius of 200 *nm*. Note that the magnetic polarizability is directly linked to the metal volume that is involved in the interaction so that the presence of the aperture will slightly decrease its value. We have verified this assumption through the determination of the polarizability of an apertureless metallic tip with radius *R* = 250 *nm* and get a value of *α*^*m*^ = −17.44 × 10^−21^ *m*^3^/*μ*_0_ instead of 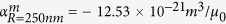 with BNA. Consequently, we stress the fact that apertures in SNOM tip are not necessary to induce the magnetic effect; simple metallic nano-rod is more efficient as already demonstrated in ref. 2.

The electric term of Eq. 1 can be determined by subtracting the pure magnetic effect of Fig. 4a, observed in the case of off-resonant BNA, from the effect corresponding to a resonantly excited BNA (keeping the tip at the same position and rotating the BNA by 90°). The obtained values are presented in Fig. 4b. Contrarily to the magnetic term, the relative shift (in logarithmic scale) of the induced electric dipole exhibits a non-linear behavior versus the BNA-to-PC distance clearly denoting the non validity of the quasi-static approximation. A maximum of redshift is obtained (see vertical dashed lines in Fig. 4b) corresponding to a critical coupling between the two resonators (i.e. maximum energy transfer between them^24^). This inevitably induces a maximum mode volume and leads to a maximum redshift of the RW. As expected, Fig. 4b shows that the more the tip radius is large, the more the tip-to-PC coupling is strong and the more the distance at which the critical coupling occurs is high. This is directly related to the electromagnetic energy redistribution inside and around the cavity induced by the presence of the tip^30^.

**Figure 5:**
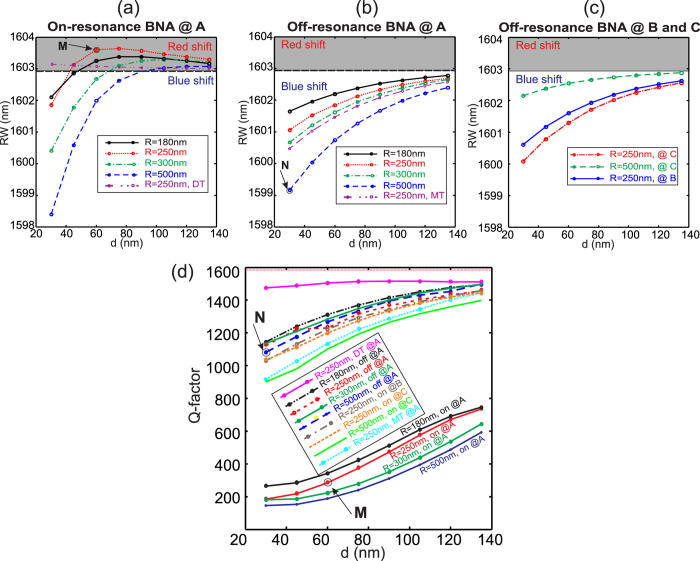
(**a**) Resonance wavelengths (RW) calculated in the case of a resonant BNA-on-tip placed above the CL7 center in position A (see white arrow in Fig. 2d) as a function of the tip-to-CL7 distance *d*. The *RW* shift is both positive and negative with respect to *d* demonstrating the double-way tunability. The case of a pure dielectric tip (similar to that of ref. 16) is also presented in dashed-dotted-dotted magenta line. (**b**) same as (**a**) in the case of non-resonant BNA. Electric effect is very weak and only blueshifts are obtained. The case of a pure metallic tip (as in ref. 2) is presented for comparison. (**c**) same as (**a**,**b**) but for tip apex positioned at the vertical of points B and C (see Fig. 2c,d). Note that the B position corresponds to a maximum of the magnetic field vertical component and a zero transversal electric field while all these three components vanish at position C. (**d**) Q-factors of the thirteen studied configurations corresponding to different apex radii (from *R* = 180 *nm* to *R* = 500 *nm*) and BNA state (on or off resonance) including uncoated dielectric tip (DT) and metallic tip (MT) as a function of the BNA-to-CL7 distance *d*. The red dashed horizontal lines at the top of the figure corresponds to the case of the unperturbed CL7 cavity (*Q*_*CL*7_ = 1590).

Other simulations were done for different configurations including both the modification of the tip position, the BNA direction (on- and off-resonance) and the tip apex radius. These configurations are compared to the case of an uncoated dielectric tip (DT) and to the case of a metallic tip (MT). The numerical results are presented in Fig. 5. Because negative and positive shifts occur, we plotted the RW values (Fig. 5a–c) instead of the relative shift presented in Fig. 4. The Q-factor variations are also given in Fig. 5d. As expected, Fig. 5a shows the competition effect between the induced electric and magnetic dipoles by the tip apex. This competition only appears when the BNA resonates (position A given by white arrow on Fig. 2d) i.e. when the antenna gap is immersed in a transverse electric field directed along its metallic arm (y-direction here). If the BNA position corresponds to a node of transverse electric field (positions B or C pointed by the green and the blue arrows respectively) or if it is off-resonance (even in the position A), only blueshifts of the RW occur. In addition, for BNA-on-tip with large apex radius, the blueshift is more pronounced meaning larger magnetic polarizability as already demonstrated above. Meanwhile, the quality factor given in Fig. 5d is significantly reduced when the BNA resonates because the stored electromagnetic energy that was confined in the CL7 cavity is now dissipated by the BNA. This leads to a decrease of the photon lifetime and thus to a decrease of the Q-factor of the cavity resonance. Similar decreasing of Q-factor was also obtained in refs 10,18 even if no electric resonance is involved (non-resonant dielectric tip).

Note that, on one hand, for a big tip with *R* = 500 *nm* (blue dashed line in Fig. 5a), the two effects (blue and red shifts) compensate for a distance *d* = 90 *nm* while this effect occurs at smaller distance (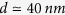) for a tip apex radius of *R* = 250 *nm* (red dotted line in Fig. 5a). Nevertheless, for both configurations, the quality factor of the whole structure decreases to 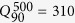 and 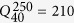 respectively compared to *Q*_*CL*7_ = 1590 of the unperturbed cavity (see Fig. 5d). Consequently, from ref. 22, we estimate that the energy dissipated by the BNA-on-tip (radiated inside the fiber and/or absorbed by the metal) is around 80% and 86% of the total energy initially stored in the cavity respectively. On the other hand, for *R* = 500 *nm*, a Δ*λ* = 5 *nm* blueshift is achieved by only approaching the BNA-on-tip at 30 *nm* from the PC. Larger shifts can occur for smaller tip-to-PC distances as already experimentally demonstrated in ref. 15 where 16 *nm* blueshift were observed for *d* = 10 *nm*. This value corresponds to an efficient tunability of the cavity resonance in comparison with the value of Δ*λ* = 1.7 *nm* that was recently obtained by focusing effect on plasmonic nanorod coupled with a PC cavity^2^.

**Figure 6:**
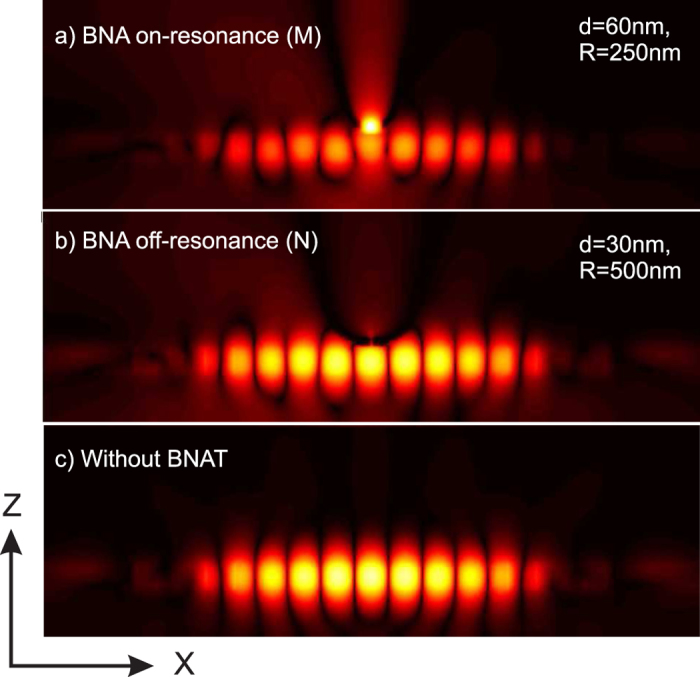
(**a**–**c**) calculated electric intensity distributions (in logarithmic scale) corresponding to the case of the two points M (in Fig. 5a,d) and N (in Fig. 5b,d) in addition to the case of the unperturbed PC cavity respectively. One can clearly see in (**a**) that the BNA pumps the PC energy and then dissipates it mainly inside the tip while the spatial extension of the cavity mode is clearly reduced in (**b**), compared to the unperturbed cavity in (**c**), due to the presence of the metal tip apex that squeezes the electromagnetic evanescent field down to the cavity. This result is consistent with the obtained discrepancy between the Q-factor of these two configurations given in Fig. 5b.

Finally, the light distribution inside the structure is shown in Fig. 6 at the resonance wavelength of the hybrid structure for two contrasting configurations indicated by the points *M* and *N* in Fig. 5a,b,d. Our main goal is to point out the spatial expansion or squeezing of the cavity mode with regard to the case of the unperturbed cavity resonance given in Fig. 6c. As it can be seen on Fig. 6a, the resonance of the BNA leads to a dramatic reduction of the light energy inside the CL7 due to a significant penetration inside the BNA gap zone (compared to the Fig. 6c). On the contrary, for an off-resonant BNA, a volume reduction of the energy distribution can be observed (see Fig. 6b) especially near the BNA-on-tip and it is accompanied by a small decrease of the photon lifetime in the CL7 cavity (slight reduction of the resonance quality factor).

## Conclusion

This study clearly demonstrated that a unique perturbation element, BNA-on-tip for instance, has the ability of red- and blue-shifting the resonance of an optical nanocavity in a fine and reversible postproduction tuning. Nevertheless, as in ref. 10, redshifts are achieved at the cost of significant Q-factor reduction. To bypass this constraint, optimization of the perturbation element design is needed in order to increase the photon lifetime inside the whole structure by decreasing both the far-field energy scattering and the metal absorption. Such perturbation elements with larger Q-factor appear to be a promising solution.” An experimental demonstration of the coupling between an active PC cavity (laser mode) and a BNA-on-tip was already performed^22^ but only blueshift of the cavity resonance was observed because the nano-antenna resonance was not as strong as predicted. This points out that the perturbation element design is critical in the achievement of the desired effect onto the cavity. Based on this configuration, alternative tip and antenna geometries - such as Diabolo nano-antennas that exhibits both electric and magnetic resonances^31^. **-** may be found to accomplish this two-way spectral tunability of a nanocavity resonance while preserving its Q-factor even in the case of redshift.

## Methods

All the numerical simulations were done with home-made Finite Difference Time Domain (FDTD) codes developed by FB. These codes include Perfectly Matched Layers as absorbing boundary conditions and a non-uniform meshing in order to faithfully describe the small features of the structure. For example, the BNA is entirely discretized with small cubes of 5 × 5 × 5 *nm*^3^ while a coarse meshing (25 × 25 × 25 *nm*^3^) is applied elsewhere. A gradual transition between the two meshing zones is respected to avoid parasitical reflections related to this spatial step change. The exploitation of the results is made under Matlab environment through codes that allow fourier transform of temporal signals and determination of Q-factor and position resonances. Figure curves and maps are also plotted with Matlab and presentations are then enhanced with CorelDRAW.

## Additional Information

**How to cite this article**: Baida, F. I. and Grosjean, T. Double-way spectral tunability for the control of optical nanocavity resonance. *Sci. Rep.* **5**, 17907; doi: 10.1038/srep17907 (2015).
